# Repurposed Automated Handheld Counter as a Point-of-Care Tool to Identify Individuals ‘At Risk’ of Serious Post-Ivermectin Encephalopathy

**DOI:** 10.1371/journal.pntd.0003180

**Published:** 2014-09-18

**Authors:** Sasisekhar Bennuru, Sébastien D. S. Pion, Joseph Kamgno, Samuel Wanji, Thomas B. Nutman

**Affiliations:** 1 National Institute of Allergy and Infectious Diseases, National Institutes of Health, Bethesda, Maryland, United States of America; 2 UMI 233, Institut de Recherche pour le Développement (IRD) and University of Montpellier, Montpellier, France; 3 Center for Research on Filariasis and other Tropical Diseases, Yaoundé, Cameroon; 4 Faculty of Medicine and Biomedical Sciences, University of Yaoundé, Yaoundé, Cameroon; 5 Research Foundation in Tropical Diseases and the Environment, Buea, Cameroon; 6 Department of Microbiology and Parasitology, University of Buea, Buea, Cameroon; University of California, San Francisco, United States of America

## Abstract

**Introduction:**

Administration of ivermectin (IVM) as part of mass drug administration (MDA) campaigns for onchocerciasis and/or lymphatic filariasis (LF) has been suspended in areas co-endemic for *Loa loa* due to severe post-treatment adverse events (SAEs) associated with high-burden of infection (>30,000 mf/ml). One simple approach for preventing SAEs is to identify and exclude individuals at risk from MDA. Here, we describe a repurposed hand-held automated cell counter (Scepter 2.0; HHAC) as a rapid, point-of-care method for quantifying microfilariae (mf) in the blood of infected individuals.

**Methodology/Principal Findings:**

The quantification of microfilarial levels in blood of naturally infected humans, experimentally infected baboons, or mf-spiked human blood was tested using a microfluidic-based automated counter and compared to traditional calibrated thick-smears. We demonstrate that mf can be quantified in 20 µl of whole blood following lysis with 10% saponin within a minute of obtaining blood. There was a highly significant concordance between the counts obtained by the HHAC and those by microscopy for mf densities of >5,000 (p<0.0001, r_c_ = 0.97) or >30,000 per ml (p<0.0001, r_c_ = 0.90). Preliminary proof of concept field studies in Cameroon with 20 µl of blood from *L. loa* infected humans (n = 22) and baboons (n = 4) also demonstrated a significantly high concordance (p<0.0001, r_c_ = 0.89) with calibrated thick blood smears counts.

**Conclusions/Significance:**

A repurposed HHAC is a portable, sensitive, rapid, point-of-care and quantitative tool to identify individuals with high levels of *L. loa* mf that put them at risk for SAEs following MDA. In addition, it provides ease of data storage and accessibility.

## Author Summary

Mass drug administration (MDA) efforts with ivermectin-based regimens for onchocerciasis and for lymphatic filariasis in Africa have been suspended in certain areas that are co-endemic for *Loa loa* infection. This is due to the serious adverse events (encephalopathy and death) that can develop following ivermectin administration in individuals with very high circulating levels of *Loa loa* microfilariae (mf) (>30,000 mf/ml). Currently thick-blood smears are performed to quantify the mf levels that are neither rapid nor high throughput. To develop a point of care (POC) strategy for rapid and high throughput mf quantification, a repurposed handheld automated counter (HHAC) was assessed and shown to be an efficient POC tool to identify individuals with very high *Loa loa* microfilaraemia in a manner sufficient to be used as part of a Test and (not) Treat (TNT) program in an effort to restart the suspended MDA programs while preventing severe adverse neurologic events.

## Introduction

Among the many parasitic helminth infections, the diseases caused by *Loa loa, Wuchereria bancrofti and Onchocerca volvulus* infections are major public health and socio-economic problems in many countries in West and Central Africa. Commonly, individuals harboring microfilariae (mf) of *W. bancrofti* and *L. loa* can have extremely high parasite burdens, but are clinically asymptomatic. This balanced host/parasite state is often perturbed when patients are treated with the antifilarial drugs ivermectin (IVM) or diethylcarbamazine (DEC) whose actions are primarily directed against the microfilarial stage of these parasites. While it is not of direct concern in this study, severe post-treatment adverse events (SAEs) have been attributed to the rapid killing of the mf that in turn is associated with inflammatory responses to the parasite and, in the case of *W. bancrofti* and *O. volvulus*, to its *Wolbachia* endosymbiont [1].

Ivermectin has been used since ∼1988 as the basis for mass treatment strategies in the control of onchocerciasis [2]–[4] and in combination with albendazole for elimination campaigns for lymphatic filariasis (LF) in Africa. Administration of IVM as part of mass drug administration (MDA) campaigns in areas co-endemic for *L. loa* resulted in SAEs with resultant encephalopathy and death in individuals harboring >30,000 *Loa loa* mf/ml of blood [5]–[8], similar to that seen in treatment of loiasis with DEC [9]. Individuals with >8000 mf of *Loa loa*/ml are also at risk for SAEs (though primarily non-neurological) that have resulted in temporary functional impairment that is usually reversible. Individuals harboring fewer than 8,000 *Loa loa* mf/ml have been considered to be at little serious risk for serious post-ivermectin adverse events. It has been estimated that approximately 5% and 1% of the ∼13 million *Loa*-infected individuals harbor >8000 mf/ml and >30000 mf/ml respectively [10].

The *Loa*-associated SAEs have led to the suspension of the MDA programs for onchocerciasis and LF in areas highly endemic for loiasis [8], [11], and this has been a major setback for LF and onchocerciasis elimination campaigns in certain West and Central African countries.

Simple and potential solutions suggested to address the prevention of *Loa*-associated SAEs have focused on “safer” treatment regimens and/or excluding those *Loa*-infected individuals at the highest risk for SAEs (those with high levels of microfilaraemia) from IVM-based MDA. Because *Loa loa* does not contain the *Wolbachia* endosymbiont, a six-week course of doxycycline, a drug that could target the *Wuchereria bancrofti* and *Onchocerca volvulus* (in Africa), would be alternative therapeutic choice. Limited data from a community-based study in an *O. volvulus*-endemic area suggest that doxycycline would be a safe regimen to treat patients co-infected with low to moderate levels of *Loa loa* (<8000 mf/ml) microfilaremia [12]. Because new drug development may take decades to be available at the community level in Africa, the objective of the present study was to develop a point-of-care diagnostic tool to identify individuals with levels of microfilaremia (>5,000 mf/ml and >30,000 mf/ml) that put them at risk for SAEs with the ultimate goal of excluding them from MDA. The small proportion of excluded individuals (∼5% of the population) could be considered for alternative treatment regimens such as 3 weeks of albendazole alone or 6 weeks of doxycycline would be safe but impractical on large-scale.

This strategy termed “Test and (Not) Treat” (TNT) relies, however, on having the tools available to perform such tests at the community level in Africa. One approach to the identification of such a tool was to re-purpose existing (on the market) devices for the rapid and accurate quantitation of mf. One such device is the Scepter 2.0 (EMD-Millipore) that is a handheld automated cell counter (HHAC) that was developed as a rapid and convenient method for enumerating purified population of cells. Here we report the adaptation of the Scepter 2.0 for use in the quantitation of mf of *L. loa* as well as for other blood-borne filarial species.

## Materials and Methods

### Parasites and Blood

Sources of mf for this study included: 1) *Brugia malayi* from peritoneal cavities of infected gerbils (*Meriones unguiculatus*) or infected blood from cats (both obtained under contract with the University of Georgia (Athens, GA); 2) *Dirofilaria immitis* infected dog blood (also from the University of Georgia, Athens, GA); or 3) *L. loa* from naturally infected humans (infected individuals from Cameroon and patients seen at the NIH) or experimentally-infected baboons (*Mandrillus* sp) in Cameroon (University of Buea). Uninfected blood from cats, dogs, or humans was used as controls.

### Ethics Statement

The human blood was obtained with informed consent from all participants under protocols approved by the IRB of the NIAID and the study was conducted on the registered protocol NCT00001230 (for the United States) and NCT01593722 (in Cameroon). Oral consent was obtained from illiterate participants in the presence of village elders as approved by the ethical review committees. The animal procedures were conducted in accordance with the guidelines with animal care and use committee at the National Institutes of Health and University of Georgia. The use of non-human primates for research was approved by the Committee on the Ethical Use of Animals in Research (CEUAR), Research Foundation for Tropical Diseases and Environment (ReFoTDe), Cameroon.

All relevant guidelines of the International Primatological Society (IPS) on the acquisition, care and breeding of non-human primates (Second Ed, 2007) for research were followed. Baboons were housed in large custom built cages that extend from floor to ceiling allowing animals to take the maximum advantage of the space available. Individual baboons were allowed to display their normal repertoire of locomotor behavior (walk, climb, run, jump and swing) by providing them with vertical climbing surfaces and perches. Horizontal surfaces were also provided to allow them to rest comfortably and perform their social interactions such as sprawling during grooming. Baboon behavior were regularly monitored to identify indications of poor welfare. Baboons received regular food and water and were fed on a broad range of food to mimic their natural diet (leaves, grass, roots, bark, flowers, fruit, lichens, tubers, seeds, mushrooms, corms, and rhizomes). They were also fed on complete commercially available diet. The health and well-being of the baboons were regularly assessed by an animal welfare officer who advised on matters such as disease prophylaxis, zoonoses, anesthesia, and methods of humane euthanasia and provision of health certificates. All measures were taken to minimize suffering during captivity and under experimentation. Health screening of workers was performed to prevent animal losses from diseases transmitted from humans to baboons as well as zoonotic transmission of disease from baboons to workers.

### Microscopy

Calibrated thick blood smears from *L. loa* infected humans and baboons were prepared and stained with Giemsa as previously described [10]. Each of 2 slides was counted twice by each of two independent readers. The geometric mean of the four readings was used.

*B. malayi* mf were purified as described earlier [13] and enumerated microscopically. For infected blood samples, 20 µl of whole blood was lysed with 80 µl of 10% saponin in a 96-well flat-bottomed plate and counted using an inverted microscope; the average of 5 readings was taken and expressed as mf/ml.

### Lysis of Blood

Lysing agents included: 1 M guanidine hydrochloride and saponin (Sigma-Aldrich, St. Louis, Mo), Lysis Buffer AL (Qiagen, Gaithersburg, MD), ACK lysing solution (Invitrogen, Carlsbad, CA), Zap-OGLOBIN II (Beckman Coulter), Sodium Dodecyl Sulfate (SDS). All agents were prepared in 0.85% NaCl (normal saline) solution.

### HHAC Counting

20 µl of blood was lysed with 80 µl of 10% saponin in a 96-well round-bottomed plate. The lysed samples were aspirated and mf were enumerated with the Scepter 2.0 (Millipore) using 60 µm sensors (Cat No. PHCC60050). Events falling in the specified mf gates were analyzed.

### Statistical Analyses

Unless otherwise stated, geometric means of microfilarial counts were used as measures for central tendency (Prism V 6.0 (GraphPad, La Jolla, CA)). Correlations and concordance between microscopic counts and those by the HHAC were assessed using the Lin's concordance correlation coefficients (http://services.niwa.co.nz/services/statistical/concordance). Specificity, sensitivity, positive predictive value (PPV) and negative predictive value (NPV) for HHAC were calculated based on comparison with microscopy.

## Results

### Adaptation of HHAC for Detecting mf

**Figure 1:**
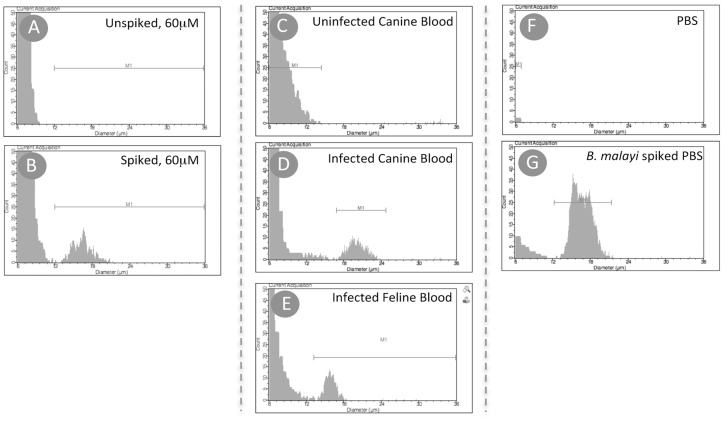
Adaptation of HHAC for detecting mf. All samples were lysed and analyzed by the HHAC using the 60 µm sensor. Histograms shown are representative samples of normal human blood before (A) and after spiking with *B. malayi* mf (B); uninfected canine blood (C), *D. immitis* infected canine blood (D) and *B. malayi* infected feline blood (E); phosphate buffer saline (PBS) before (F) and after spiking with *B. malayi* mf (G).

Preliminary studies demonstrated the need to lyse the blood sample before analyzing it using the 60 µm sensors (**Text S1**). Saponin-based lysis was found to be rapid and performed in small volumes (100 µl-final volume). Using a lysis procedure in which 20 µl of mf containing blood was lysed with 80 µl of 10% saponin resulted in an easily identifiable peak (12–20 µm) using the 60 µm sensor. The corresponding peaks were absent in normal unspiked human blood (**Figure 1A, 1B**). Lysis of the cat or dog blood (experimentally infected with *B. malayi* (in cats) and *D. immitis* (in dogs)) and analysis using a 60 µm sensor displayed distinct peaks (18–24 µm, *D. immitis*; 12–20 µm, *B. malayi*) in the infected blood samples, suggesting that the events being counted were indeed mf (**Figure 1C-1E**).

### Confirmation of Microfilarial Peaks

To confirm that the “events” counted were truly mf, we used PBS spiked with purified *B. malayi* mf. As shown in **figure 1F**, there was no detectable peak (12–24 µm) in control PBS alone whereas there was an easily identifiable peak in the mf-spiked PBS (**Figure 1G**). Furthermore, examination of the sensor using an inverted microscope indicated that the mf could easily be seen passing through the 60 µm microfluidic system without clogging the aperture (**Figure S1 in Text S1**).

### Noise and Optimization for Quantification of mf

**Figure 2:**
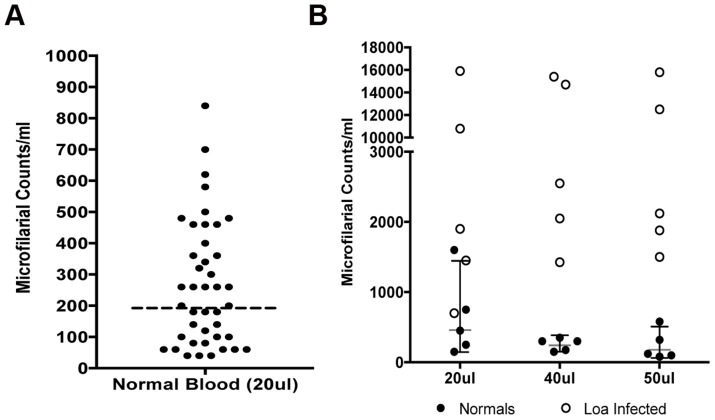
Signal to noise in HHAC. HHAC counts of uninfected normal human blood bank donors using 20 µl of blood samples (A). Increasing the blood volume (40 µl or 50 µl) does not change the noise (normal blood, solid dots) compared to *L. loa* microfilarial counts (open circles)(B).

Although there was no detectable peak visually in the normal human blood or PBS (**Figure 1A & 1F**), an inherent electronic signal (noise) resulted in spurious low number of events in the gated area (12–36 µm) that ultimately influenced the final “counts”. Analyses of 20 µl of whole blood from several healthy, uninfected donors showed a range of background electrical signals resulting in noise that (when calculated) would have provided counts that ranged between 40–840 mf/ml (Geometric mean (95% CI): 192.2 (146.4–252.4)) (**Figure 2A**). Increasing the volume of blood (40–50 µl) did not alter the background noise or the true mf counts (**Figure 2B**) suggesting that the “noise” in the system was emanating from the device rather than from the blood sample. Further testing with 5–50 µl of *B. malayi* infected cat blood resulted in similar counts after adjusting for dilution factor, suggesting that volume of blood is not a limiting factor (**Figure S2 in Text S1**). As expected there was an increase in size of histogram peaks generated proportional to the volume of sample.

Because the off-the-shelf programming of the HHAC is based on a 50 µl sample containing purified human cells, an algorithm was created to take into account the noise, dilution factor and the initial blood volume for enumerating mf. Assuming uniform distribution of mf, to obtain the final mf count (mf/ml), the background “noise” was subtracted and then multiplied by the dilution factor (x 5).

### Laboratory Testing: Sensitivity and Specificity

**Figure 3:**
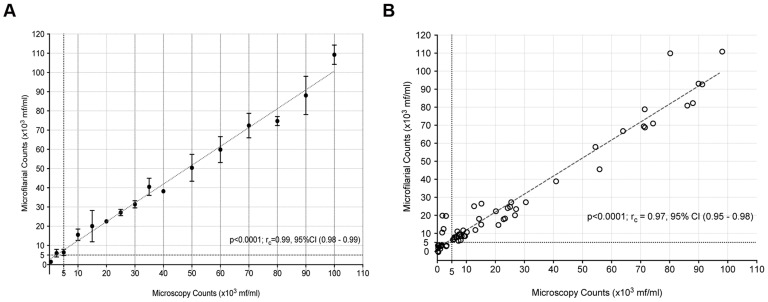
Correlation of HHAC counts with microscopy counts of mf. Purified mf of *B. malayi* spiked in normal human blood was tested in triplicate with a 60 µm sensor at various concentrations (0 to 100,000 mf/ml): (A) serially diluted from 100,000 mf/ml or (B) blinded at various random concentrations. Microscopy counts* plotted on the x-axis and HHAC counts in triplicate on y-axis (x10^3^ mf/ml). ‘r_c_’ denotes concordance correlation coefficient. * - Counts based on serial dilution.

A simplified one-step lysis procedure (20 µl of whole blood +80 µl of 10% saponin) mixed in a well of a 96-well round bottomed plate was found to be convenient, rapid, and to give reproducible results. To evaluate the ability of this simplified procedure to enumerate mf, microscopically enumerated *B. malayi* mf were spiked in normal human blood at concentration of 100,000 mf/ml and tested in triplicate determinations at dilutions that varied from 500 mf/ml to 100,000 mf/ml. As shown in **figure 3A**, there was extremely good concordance correlation coefficients between the counts obtained by the HHAC (p<0.0001; r_c_ = 0.95, CI (0.83–0.98) and those based on microscopy.

Further, the HHAC was tested in a blinded fashion with *B. malayi* mf spiked samples. Four mf density ranges (1–5,000 mf/ml; 5001–10,000 mf/ml; 10,001–30,000 mf/ml and 30,001–100,000 mf/ml) were tested, with each range consisting of 15 randomly generated numbers. As expected and in accordance with the need for identifying individuals harboring greater than 5,000 mf/ml, highly significant concordance correlation coefficients were observed (p<0.0001, r_c_ = 0.97, CI (0.96–0.98)). The concordance coefficient correlations for mf levels greater than 30,000 mf/ml were also observed to be highly significant (p<0.0001; r_c_  = 0.90, CI (0.76–0.96)) (**Figure 3B**). However, the ability of the HHAC to reliably detect mf at densities below 5000 mf/ml is poor (p = 0.02; r_c_ = 0.23, CI (−0.002–0.44)) that is partly dependent on the background noise in the system.

Overall, under laboratory setting, the HHAC was observed to be highly efficient for enumerating microfilarial counts at densities >30,000 mf/ml and >5,000 mf/ml with positive predictive values of 93% and 91% respectively in 20 µl of whole blood (**Table 1**).

**Table 1:** Performance of HHAC under laboratory and field conditions.

	*B. malayi (mf/ml)*		*L. loa (mf/ml)*	
	>5000	>30,000	>5000	>30,000
**Specificity**	0.73 (0.44–0.92)	0.97 (0.88–0.99)	0.85 (0.42–0.99)	0.95 (0.76–0.99)
**Sensitivity** *	0.91 (0.80–0.97)	1.0 (0.76–1.0)	0.95 (0.75–0.99)	0.66 (0.22–0.95)
**PPV**	0.91 (0.80–0.97)	0.93 (0.68–0.99)	0.95 (0.75–0.99)	0.80 (0.28–0.99)
**NPV**	0.73 (0.44–0.92)	1.0 (0.92–1.0)	0.85 (0.42–0.99)	0.90 (0.70–0.98)

*versus microscopy; PPV: Positive Predictive Value; NPV: Negative Predictive Value;

Values in brackets denote 95% CI.

### Field Testing: Sensitivity and Specificity

**Figure 4:**
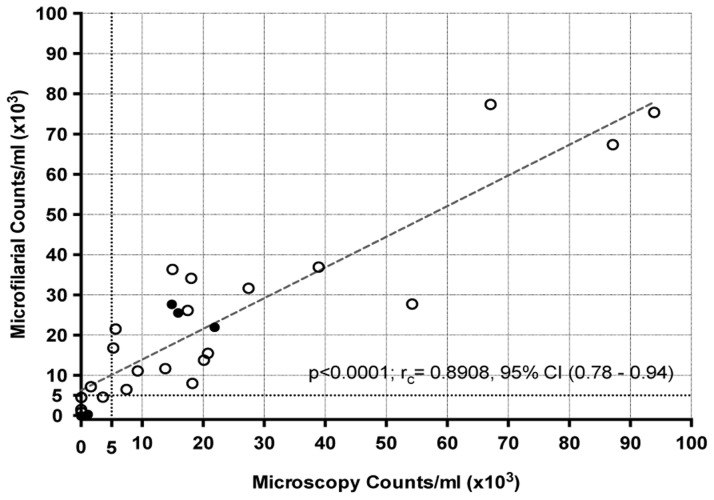
Correlation of HHAC counts with microscopy counts of *L. loa* mf. Field-testing of *L. loa* infected human (open circles) and baboon (solid dots) blood counts by HHAC compared with calibrated thick-smear microscopic counts.

Field-testing of the HHAC was carried out with baboons experimentally infected with *L. loa* (n = 4) blood and *L. loa* infected individuals (n = 22) from Cameroon as preliminary ‘proof of concept’ study; parallel calibrated thick blood smears were used for comparison. As shown in **Figure 4**, there was significant concordance (p<0.0001; r_c_ = 0.89, CI (0.78–0.94)) between the counts obtained by the HHAC and those of the calibrated thick-blood smear. As shown in **Table 1**, for *L. loa* microfilaremia detection, the positive predictive values were 95% for mf densities >5000 mf/ml and 80% for levels >30,000 mf/ml. It should be noted that while the data obtained from the HHAC occurred within minutes of obtaining the blood, the time to result for the calibrated thick smear ranged from 4 hours to several days. Although there was a highly significant correlation, it was apparent that the HHAC performed less well when mf counts were <5000/ml.

## Discussion

Areas co-endemic for *L. loa* and either *O. volvulus* or *W. bancrofti* pose a challenge for MDA programs throughout Central and West Africa. Since post-IVM SAEs are associated with high-levels of circulating *L. loa* mf, there is a need for a point-of-care tool to identify these individuals at highest risk (>30,000 mf/ml) for the development of life-threatening SAEs. Currently, calibrated thick smears of whole blood are used as the ‘gold’ standard to quantify mf. The major disadvantage of this system is the time it takes between the collection of blood and the time to a result, a time that includes drying the slide (1–2 hours), fixation, staining the slide (typically 1 hour) and reading 2 slides per patient (up to a half an hour per slide if levels are high). Thus, for microscopy, the time between blood collection and microfilarial quantification can range from 4 hours to several days depending on ambient temperature and humidity. Although thick blood smears, in general, provide better sensitivity for low levels of microfilaraemia, in situations of very high mf loads discrepancies in quantification arise because of the difficulty in quantifying overlapping (or knotted) mf. Similarly, PCR based diagnostic methods [14] and loop-mediated isothermal amplification (LAMP) assays that are sensitive and semi-quantitative [15], or immunoassays that are only qualitative require a laboratory-based setting an, and to date are not point-of-care or field-friendly.

Field applicability for point-of-care diagnostics requires a simple, quick, inexpensive and reliable methods that would allow, in the case of the TNT strategy for the precise “on the spot“ identification of those individuals with mf >30000 mf/ml. The HHAC is an automated hand held counter that uses a microfluidic based-sensor for counting and sizing particles based on the coulter principle, where the electric pulses correspond to the count of particles passing through and their amplitudes to their volumes. However, the HHAC sensors are technically limited by cell numbers and hence not amenable to whole blood analysis (**Text S1**). Hypothesizing that by removing RBC's from the whole blood, we can overcome the limitation of the system, lymphocytes and mf can then be visualized and distinguished from one another based on size. Microfilariae in general, range from 3-7 µm in diameter and 160–300 µm in length. Additionally, in contrast to the conventionally measured spherical shaped cells, the mf could be passing through the microfluidic sensors' aperture in any possible conformation (linear, curled, bent etc.). Although not currently able to explain the difference in the gated size between the WBCs (<12 µm) and the mf (>12 µm) it is nevertheless clear that these 2 components of the lysed whole blood could be easily separated (**Figure 1**).

The HHAC seems to be able to differentiate between *B. malayi* (∼12–18 µm), *Loa loa* (∼15–20 µm) and *D. immitis* (∼18–24 µm) by their average ‘diameter’ in the histogram (**Figure S3 in Text S1**). Though its efficiency in discriminating amongst the other blood-borne filarial pathogens (*W. bancrofti, L. loa* and *M. perstans*) found in Central and West Africa needs to be evaluated, the likelihood of detecting significant numbers of *W. bancrofti* (a nocturnally periodic filarial parasite) on noontime sampling of the diurnally periodic *L. loa* is miniscule and for *M. perstans*, counts rarely exceed 1000 mf/ml.

Theoretically, increasing the volume of infected blood sample should increase the actual number of events and hence increase the signal while the noise remains constant. It is necessary to note that the increased volumes are only beneficial for increasing the sensitivity at the very low-end detection of microfilaraemia (<5,000 mf/ml) and not a necessity for the purpose of “Test and Not Treat” strategy, as the HHAC performs very well at enumerating mf >5,000 per ml of blood.

Compared to the lab-based testing with *B. malayi*, the slight drop in field performance at >30,000 mf/ml (66% sensitivity, 80% PPV) is probably due to the fact that only four individuals of those tested had microfilarial loads >30,000 mf/ml. Despite the discrepancy of the lone high-burden *L. loa* mf counts (>30,000 mf/ml), the HHAC still would positively predict (95%) >5000 mf/ml and hence re-testing before administering IVM could be considered. Together, the data from this proof-of-concept' study suggests that the HHAC can function extremely well in rapidly quantifying circulating microfilarial load and is efficient at identifying individuals at risk of SAEs. Moreover, the time from sample collection to result was less than 2 minutes.

Inherent to all diagnostic tools, the HHAC has it's own limitations. The major limitation currently is the cost associated with the sensors (approx. US$2.00 wholesale) and the initial cost of the HHAC (discounted price of ∼$2000). However, the benefits (point-of-care test, high-throughput, result to clinical decision, no cold chain) of the re-purposed HHAC might outweigh relatively lower per test costs of microscopy and PCR based methods). Indeed, it remains to be seen if the efforts being made for HHAC availability at better pricing in resource limited parts of the world helps. First, the “off the shelf” HHAC is a sensitive tool to identify *L. loa* infected individuals that harbor >30,000 mf/ml for the MDA program in West and Central Africa. Quantification of the very low levels (<5,000 mf/ml) of microfilaremia would require firmware modifications. Secondly, the time duration to positive test (>30,000 mf/ml and ‘No Treatment’) is less than two minutes from the time of blood draw (finger prick). As the device is rechargeable and battery-operated, not affected by temperature or humidity and is associated with portability of the instrument and data storage, it is highly amenable to field conditions in rural settings of West and Central Africa where it could be used.

### Conclusion

Access to rapid point-of-care diagnostic technologies in resource-limited settings imposes unique challenges [16]. While the criteria for an ideal point-of-care test can vary based on the disease setting, the function of the test or device, and also the circumstances under which test or device is implemented, the outcome should lead to expedited clinical decision making [17]. Fundamental criteria for a successful point-of-care test are its accuracy and reliability. In this context of *Loa loa* infection (in regions where onchocerciasis and/or lymphatic filariasis is co-endemic) we demonstrate that a repurposed HHAC can rapidly and reliably quantify microfilarial loads that put people “at risk” for post-ivermectin SAEs. Since the goal of point-of-care test is also to expedite the clinical decision for safe patient outcomes this on-the-spot determination of risk could help to mobilize the MDA campaigns to eliminate LF and onchocerciasis in West and Central Africa. Finally, although the advantages and efficacy of the HHAC make it a functional point-of-care diagnostic tool, a comprehensive cost analysis will be necessary to help establish whether the introduction of this tool would be worth the investment of resources for a better clinical outcome.

## Supporting Information

Text S1 **Supplemental Figures.** Figure S1 in Text S1: Microfilaria in 60 µm sensor. Center image showing the 60 µm sensor with the various ‘visible’ parts labeled 1–5 in order of the sample flowing through the microfluidic sensor. 1- Mesh to filter clumps and large particles that would block the aperture. Phase contrast microscopic images of filter focused in front of the filter (1A) and behind the filter (1B). 2- The aperture area, with phase contrast images of the triangular segment at 10x magnification (2A) and 40x magnification (2B); and 40X magnification of the banded area (2C). Microfilariae after passing through (3) and just before the instrument beeps (4) Accumulation of mf at the end of the microfluidic chamber (5A & 5B). Figure S2 in Text S1: Volume of blood sample. Histograms on the top correspond to *B. malayi* infected cat blood tested at various volumes (50 µl to 5 µl, in intervals of 5 µl). The HHAC counts corrected for the dilution factor are tabulated. M1 denotes the peak on the histogram. Figure S3 in Text S1: Size based differentiation capability of Scepter. Overlapping histograms showing the gates of *Brugia malayi* (O1, violet), *Loa loa* (O2-field, red; O3-lab, orange), *Dirofilaria immitis* (O4, blue) compared to normal human blood (O5, grey). The O5 gate corresponds to all the cells in the blood (minus RBC).
(DOCX)
