# Supplementary material for: Repurposed Automated Handheld Counter as a Point-of-Care Tool to Identify Individuals ‘At Risk’ of Serious Post-Ivermectin Encephalopathy
Source: PLoS Negl Trop Dis. 2014 Sep 18;8(9):e3180. doi: 10.1371/journal.pntd.0003180 (PMC4169247; doi:10.1371/journal.pntd.0003180)

**Repurposed Automated Handheld Counter as a Point-of-Care Tool to Identify Individuals ‘At Risk’ of Serious Post-Ivermectin Encephalopathy**

Sasisekhar Bennuru^1^, Sébastien D.S. Pion^2,3^, Joseph Kamgno^3,4^, Samuel Wanji^5,6^ and Thomas B. Nutman^1^

^1^ National Institute of Allergy and Infectious Diseases, National Institutes of Health, Bethesda, MD USA;

^2^ UMI 233, Institut de Recherche pour le Développement (IRD) and University of Montpellier, Montpellier, France;

^3^ Center for Research on Filariasis and other Tropical Diseases, Yaoundé, Cameroon;

^4^ Faculty of Medicine and Biomedical Sciences, University of Yaoundé, Cameroon;

^5^ Research Foundation in Tropical Diseases and the Environment, Buea, Cameroon.

^6^ Department of Microbiology and Parasitology, University of Buea, Cameroon

**Supplemental Data:**

*Working principle of Scepter and its exploitation*

The Scepter 2.0 is an automated hand held counter that uses a microfluidic based-sensor for counting and sizing particles. It is dependent on the electrical impedance produced as the particles/cells pass through a small aperture in a conductive liquid. The resulting electric pulses and their amplitudes correspond to the count of particles and their volumes respectively. The pulse generated is larger when multiple particles happen to pass through the aperture at the same time, and results in low particle count and higher volume measurements. The counting system is highly sensitive to the conductive properties of the diluent. High concentrations of salt (>5% NaCl) increase the conductivity but also increase the noise in the system. Optimal resistivity of 1Ω/cm is achieved with 1% NaCl. Technically, the Scepter 2.0 is not amenable (as per manufacturer’s instructions) to whole blood analysis as the sensors have been designed for purified populations of cells and are limited to cell concentrations of 50,000-1,500,000 cells/ml (40 μm sensor) and 10,000-500,000 cells/ml (60 μm sensor). Hypothesizing that by removing RBC’s from the whole blood, then lymphocytes and mf can be visualized and distinguished from one another based on size. Microfilariae in general, range from 3-7 μm in diameter and 160-300 μm in length. Additionally, in contrast to the conventionally measured spherical shaped cells, the mf could be passing through the microfluidic sensors’ aperture in any possible conformation (linear, curled, bent etc.).

**Supplemental Figures**

Figure S1: Microfilaria in 60 μm sensor. Center image showing the 60 μm sensor with the various ‘visible’ parts labeled 1-5 in order of the sample flowing through the microfluidic sensor. 1- Mesh filter to filter out clumps and large particles that would block the aperture. Phase contrast microscopic images of filter focused in front of the filter (1A) and behind the filter (1B). 2- The aperture area, with phase contrast images of the triangular segment at 10x magnification (2A) and 40x magnification (2B); and 40X magnification of the banded area (2C). Microfilariae after passing through (3) and just before the instrument beeps (4). Accumulation of microfilariae at the end of the microfluidic chamber (5A & 5B).


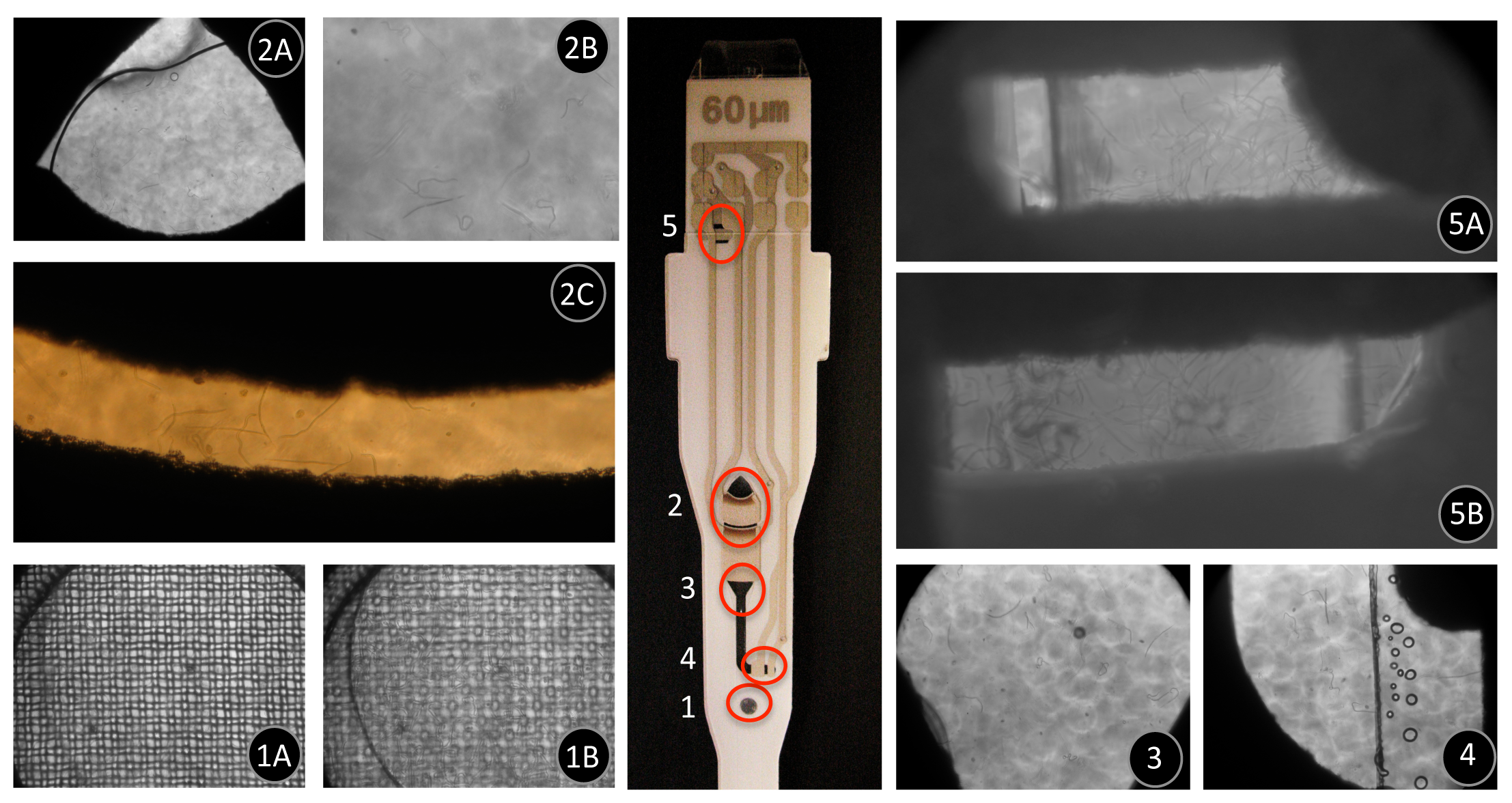


Figure S2: Volume of blood sample. Histograms on the top correspond to *B. malayi* infected cat blood tested at various volumes (50 μl to 5 μl, in intervals of 5 μl). The scepter counts corrected for the dilution factor are tabulated. M1 denotes the peak on the histogram.


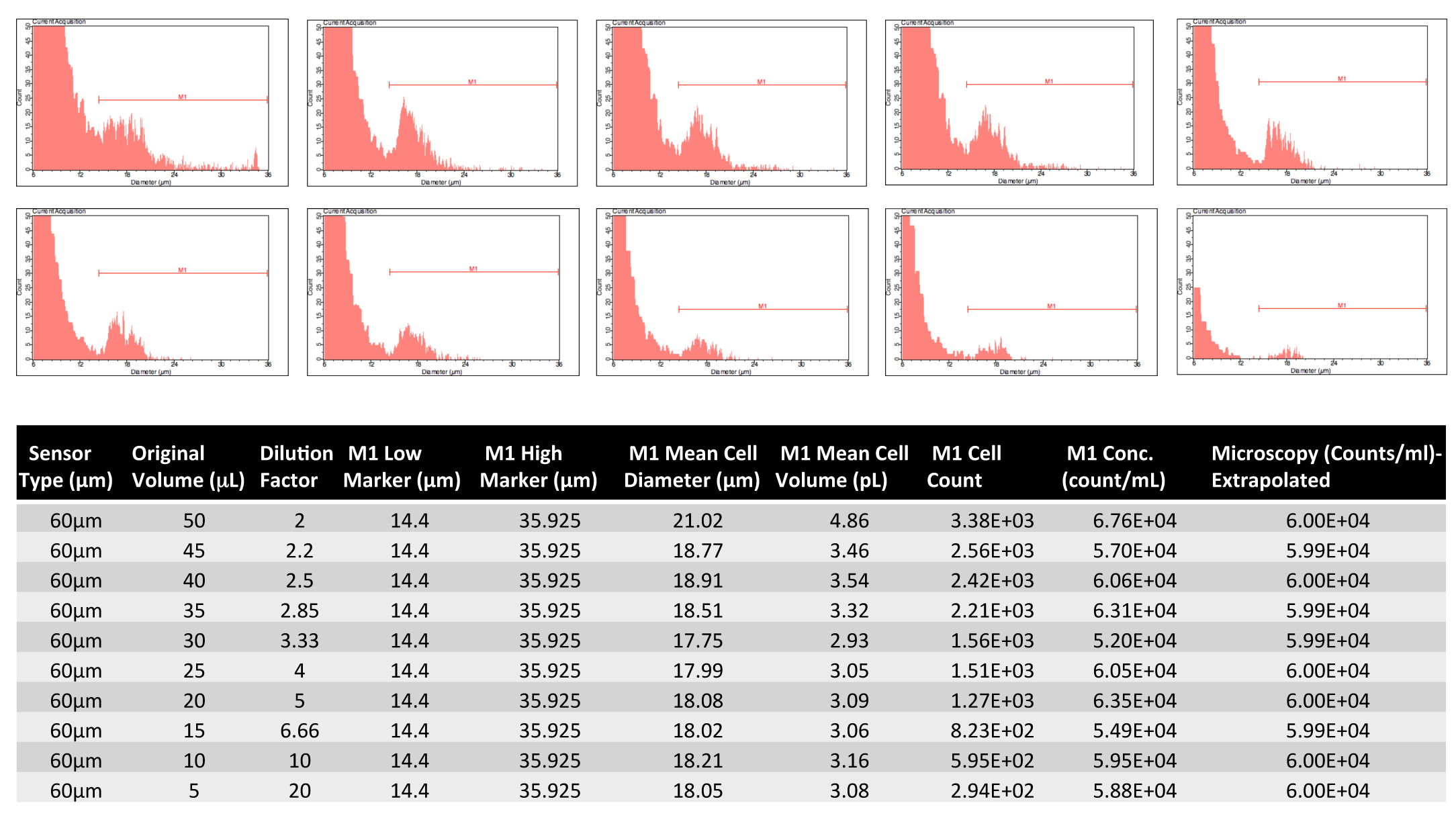


Figure S3

Size based differentiation capability of Scepter: Overlapping histograms showing the gates of *Brugia malayi* (O1, violet), *Loa loa* (O2-field, red; O3-lab, orange), *Dirofilaria immitis* (O4, blue) compared to normal human blood (O5, grey). The O5 gate corresponds to all the cells in the blood (minus RBC).


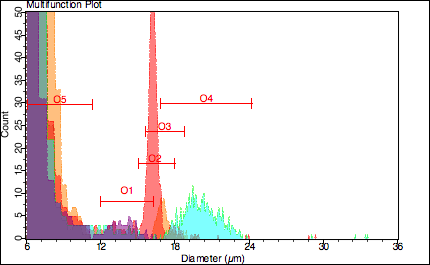

Supplement: Text S1 — Supplemental Figures. Figure S1 in Text S1: Microfilaria in 60 µm sensor. Center image showing the 60 µm sensor with the various ‘visible’ parts labeled 1–5 in order of the sample flowing through the microfluidic sensor. 1- Mesh to filter clumps and large particles that would block the aperture. Phase contrast microscopic images of filter focused in front of the filter (1A) and behind the filter (1B). 2- The aperture area, with phase contrast images of the triangular segment at 10x magnification (2A) and 40x magnification (2B); and 40X magnification of the banded area (2C). Microfilariae after passing through (3) and just before the instrument beeps (4) Accumulation of mf at the end of the microfluidic chamber (5A & 5B). Figure S2 in Text S1: Volume of blood sample. Histograms on the top correspond to B. malayi infected cat blood tested at various volumes (50 µl to 5 µl, in intervals of 5 µl). The HHAC counts corrected for the dilution factor are tabulated. M1 denotes the peak on the histogram. Figure S3 in Text S1: Size based differentiation capability of Scepter. Overlapping histograms showing the gates of Brugia malayi (O1, violet), Loa loa (O2-field, red; O3-lab, orange), Dirofilaria immitis (O4, blue) compared to normal human blood (O5, grey). The O5 gate corresponds to all the cells in the blood (minus RBC). (DOCX) [file pntd.0003180.s001.docx]
